# U. S. V. M. A.

**Published:** 1896-04

**Authors:** 


### SOCIETY PROCEEDINGS.

### U. S. V. M. A.

MEMBERS desiring to present papers before the U. S. V. M. A. at Buffalo should at once notify the Secretary, Dr. S. Stewart, Kansas City, Kan., in order that they may be assigned place in the programme, which will be issued earlier than in former years.

We publish in this number the officers, committees, secretaries, etc., so far as completed, of our national association, so that all those charged with special lines of work may find a ready reference to those from whom much will be expected at our Annual Convention at Buffalo, on September 1st, 2d, and 3d, and in conjunction therewith the New York State meeting on September 4th and 5th. In all probability the Ohio State Association will join with the National organization, with their annual gathering, having perhaps a single session of their own organization for special work of their own State. The most favorable comments from the Buffalo papers have already been accorded the Comitia Minora in selecting Buffalo as the convention city for 1896.

The Genesee Hotel has been selected by the local committee of the U. S V. M. A. as headquarters for the members. This hotel, centrally located, has been recently entirely remodelled, and the present managers are famous for their ability as caterers to the public needs and as entertainers of large gatherings. The hotel has kindly volunteered the use of a very large room for the use of the Convention, a room used before by the State Association and Association of Faculties. Should this room not prove adequate, a room at the Buffalo University has been kindly tendered and will afford ample accommodations for all, and is quite accessible to the hotel and from the centre of the city.

Messrs. Hinkley, Wende, and Willganz have called to their aid a large auxiliary committee of local veterinarians and a number of meetings have already been held. A visit to Niagara Falls is under consideration. The banquet has been provided for and a fee within the reach of all has been fixed. A plan that has worked admirably in other conventions has been considered by the Committee of providing a mid-day luncheon, which contributes so much to prompt sessions and a goodly attendance at each roll-call.

The outlook for the most successful meeting in the history of the Association is auspicious, and that there can be no failure where such energy and zeal are displayed by a local meeting goes without saying. The Committee rightly asks for a great outpouring of the members, and the JOURNAL raises its voice in appeal and will, with each issue from now until September, keep the profession posted on all matters pertaining to the coming convention.

*Officers and Committees 1895–96.* President, W. Horace Hoskins, 3452 Ludlow St., Philadelphia. Vice-Presidents, F. H. Osgood, 50 Village St., Boston, Mass.; C. C. Lyford, 821 Third Ave., Minneapolis, Minn.; R. H.

Harrison, 406 Utah Ave., Atchison, Kansas. Secretary, S. Stewart, 7½ St. James St., Kansas City, Kansas. Treasurer, James L. Robertson, 409 Ninth Ave., New York City.

Comitia Minora: Chairman, W. L. Williams, Bozeman, Montana; D. E. Salmon, Bureau of Animal Industry, Washington, D. C.; Leonard Pearson, 2024 Pine St., Philadelphia; Nelson P. Hinkley, 395 Ellicott St., Buffalo, N. Y.; L. H. Howard, 67 West Newton St., Boston, Mass.; T. B. Rayner, Chestnut Hill, Philadelphia; T. J. Turner, Columbia, Mo.

Intelligence and Education: Chairman, A. W. Clement, 916 Cathedral St., Baltimore, Md.; John Faust, Poughkeepsie, N. Y.

Diseases: Chairman, S. J. J. Harger, 205 North Twentieth St., Phila., Pa.; W. B. Niles, Ames, Iowa; C. A. Cary, Auburn, Alabama; John Wende, 1593 Main St., Buffalo, N. Y.

Army Legislation: J. P. Turner, Fort Meyer, Va.; John R. Hart, 2577 Amber St., Phila.; F. H. Mackie, Fair Hill, Md.

Publication: Chairman, W. L. Williams, Bozeman, Montana; S. Stewart, Kansas City, Kansas; Nelson P. Hinkley, 395 Ellicott St., Buffalo, N.Y.

Local Committee of Arrangements: Chairman, Nelson P. Hinkley, Buffalo, N. Y.; C. J. Willganz, 215 Carolina St., Buffalo, N. Y.; John Wende, 1593 Main St., Buffalo, N. Y.

Resident State Secretaries: Alabama, C. A. Cary, Auburn; Arizona, D. Lemay, Fort Grant; Arkansas, R. R. Dinwiddie, Fayetteville; California, R. A. Archibald, San Francisco; Colorado, J. K. Thompson, Pueblo; Connecticut, R. P. Lyman, Hartford; Delaware, H. P. Eves, Wilmington; Washington, D. C, J. H. Adamson; Illinois, R. P. Steddom, Galesburg; Iowa, G. A. Johnson, Sioux City; Kansas, N. S. Mayo, Manhattan; Louisiana, W. H. Dalrymple, Baton Rouge; Maine, H. H. Choate, Lewiston; Maryland, W. C. Seigmund, Baltimore; Massachusetts, Madison Bunker, Newton; Michigan, S. Brenton, Detroit; Minnesota, M. H. Reynolds, St. Anthony Park; Missouri, J. M. Phillips, St. Louis; Montana, W. L. Williams, Bozeman; Mississippi, Tait Butler, Agricultural College; Nebraska, A. F. Peters, Agricultural Experimental Station; New Hampshire, L. Pope, Portsmouth; New Jersey, J. P. Lowe, Passaic; New York, Claude D. Morris, Pawling; North Dakota, T. D. Hinebauch, Fargo; Ohio, J. C. Meyer, Jr., Cincinnati; Pennsylvania, W. L. Rhoads, Lansdowne; Rhode Island, J. A. McLaughlin, Providence; South Carolina, Benjamin McInnes, Charleston; South Dakota, M. J. Treacy, Fort Meade; Tennessee, J. W. Schiebler, Memphis; Virginia, George C. Faville, Norfolk; Washington, S. B. Nelson, Pullman; West Virginia, L. N. Reefer, Wheeling; Wisconsin, G. Ed. Leech, Milwaukee.

